# Cellular and Molecular Biomarkers Indicate Premature Aging in Pseudoxanthoma Elasticum Patients

**DOI:** 10.14336/AD.2019.0610

**Published:** 2019-06-05

**Authors:** Janina Tiemann, Thomas Wagner, Olivier M Vanakker, Matthias van Gils, José-Luis Bueno Cabrera, Bettina Ibold, Isabel Faust, Cornelius Knabbe, Doris Hendig

**Affiliations:** ^1^Institut für Laboratoriums- und Transfusionsmedizin, Herz- und Diabeteszentrum Nordrhein-Westfalen, Universitätsklinik der Ruhr-Universität Bochum, Bad Oeynhausen, Germany; ^2^Center for Medical Genetics, Ghent University Hospital, Ghent, Belgium; ^3^Haematology Department, Hospital Universitario Puerta de Hierro-Majadahonda, Majadahonda, Spain

**Keywords:** pseudoxanthoma elasticum, aging, CCL11, GDF11, IGF1, IGFBP

## Abstract

The molecular processes of aging are very heterogenic and not fully understood. Studies on rare progeria syndromes, which display an accelerated progression of physiological aging, can help to get a better understanding. Pseudoxanthoma elasticum (PXE) caused by mutations in the *ATP-binding cassette sub-family C member 6* (*ABCC6*) gene shares some molecular characteristics with such premature aging diseases. Thus, this is the first study trying to broaden the knowledge of aging processes in PXE patients. In this study, we investigated aging associated biomarkers in primary human dermal fibroblasts and sera from PXE patients compared to healthy controls. Determination of serum concentrations of the aging biomarkers eotaxin-1 (CCL11), growth differentiation factor 11 (GDF11) and insulin-like growth factor 1 (IGF1) showed no significant differences between PXE patients and healthy controls. Insulin-like growth factor binding protein 3 (IGFBP3) showed a significant increase in serum concentrations of PXE patients older than 45 years compared to the appropriate control group. Tissue specific gene expression of GDF11 and IGFBP3 were significantly decreased in fibroblasts from PXE patients compared to normal human dermal fibroblasts (NHDF). IGFBP3 protein concentration in supernatants of fibroblasts from PXE patients were decreased compared to NHDF but did not reach statistical significance due to potential gender specific variations. The minor changes in concentration of circulating aging biomarkers in sera of PXE patients and the significant aberrant tissue specific expression seen for selected factors in PXE fibroblasts, suggests a link between ABCC6 deficiency and accelerated aging processes in affected peripheral tissues of PXE patients.

Aging is a heterogeneous process which is characterized by a time-dependent decline of physiological functionality and integrity. Thus, as an organism ages, it gets progressively vulnerable to various pathologies like cancer, diabetes as well as cardiovascular and neurodegenerative diseases ultimately leading to death (reviewed in [1]).

Due to the complexity of aging, finding appropriate study models is still a problem. In many cases rare progeria syndromes like the Hutchinson-Gilford-progeria syndrome (HGPS) or related cell culture models displaying an accelerated aging process are used for gerontological studies [2-4]. As one single model cannot provide an entire description of potential physiological aging processes, the need for further models is steadily of great interest.

Pseudoxanthoma elasticum (PXE) is a rare inherited genetic disorder caused by mutations in the *ATP-binding cassette subfamily C member 6* (*ABCC6*) gene which causes a deficiency of the encoded transporter protein [5, 6]. In the general population the prevalence of PXE is supposed to be between 1:25 000 and 1:100 000. Females seem to be affected more often than males for yet unknown reasons (summarized in [7]). Thusfar, the molecular characteristics of PXE have only barely been discussed or interpreted in the light of premature aging processes although patients display clinical and molecular characteristics of premature aging and known premature aging syndromes. PXE patients display soft tissue calcification affecting the skin, eyes and the cardiovascular system. It primarily leads to the fragmentation of elastic fibers and remodeling of the extracellular matrix resulting in a premature loss of skin elasticity, extensive wrinkle formation, arteriosclerosis and visual impairment with similarities to macular degeneration, all of which are clinical characteristics of the elderly (summarized in [7, 8]). Molecular studies showed that ABCC6 deficiency leads to aberrant pyrophosphate homeostasis, a known calcification inhibitor, characterized by a decreased plasma concentration [9-12]. Similar observations were made in patients suffering from the premature aging syndrome HGPS [13]. It was further shown that the progression of HGPS can be decelerated by administration of statins and bisphosphonates [3]. Studies demonstrated that this therapy could be also beneficial for PXE although the underlying molecular mechanisms remain unclear [14-16].

Additionally, it was shown that ABCC6 deficiency leads to an aberrant mitochondrial function and a loss of proteostasis characterized by abnormal expression and serum concentrations of matrix metalloproteinases [17, 18]. Both, mitochondrial dysfunction and a loss of proteostasis are known to play a role in physiological aging [19-21].

Additionally, studies showed that blood from young mice have rejuvenating effects in old mice, demonstrating that specific blood factors need to change concentrations during the course of aging [22-24]. Similar assumptions could be made for PXE pathogenesis as parabiosis experiments with *Abcc6^-/-^/ Rag^-/-^* and wild-type mice resulted in a prevention of further ectopic mineralization in *Abcc6^-/-^/ Rag^-/-^* mice [25]. Furthermore, it was shown that normal human fibroblasts form abnormal elastic fiber aggregates when cultured with serum from PXE patients [26].

To gain insight into potential blood factors involved in PXE pathogenesis, the investigation of aging processes in PXE patients by determination of biomarkers showing clear association with aging could be helpful. Changes in concentration of proteins like growth differentiation factor 11 (GDF11), eotaxin-1 (CCL11), insulin-like growth factor 1 (IGF1) and its binding protein insulin-like growth factor binding protein 3 (IGFBP3) were associated with aging processes [24, 27-30]. By analyzing these aging biomarkers in sera and fibroblasts of PXE patients, we could show aberrant mRNA and protein expression in supernatants of PXE fibroblasts as well as abnormal serum concentrations of specific aging biomarkers in PXE patients underlining the suggestion of potential premature aging processes. With this, we were able to give first evidence for a link between an ABCC6 deficiency and accelerated aging processes especially in affected peripheral tissues of PXE patients.

## MATERIAL AND METHODS

### Experimental Design

The study was approved by the ethics commission of the Ruhr University of Bochum Faculty of Medicine, located in Bad Oeynhausen. Patients participating in the study gave their written informed consent.

The goal of the study was to investigate known aging biomarker in PXE sera to evaluate whether potential premature aging processes contribute to PXE pathogenesis. Because little is known about aging processes of PXE patients, some of the most prominent serum proteins with clear connection to aging (CCL11, GDF11, IGF1 and IGFBP3) were chosen to get first insights [24, 27-30]. In case of ambiguous results or aberrant serum concentrations in PXE patients compared to controls additional mRNA analysis in fibroblasts and protein determination in cell culture supernatants was performed when possible.

### Patient characteristics

In all patients the diagnosis of PXE was consistent with the consensus criteria reported [31]. Age/sex-matched blood donors served in this study as healthy controls. For analysis, each cohort was divided into subgroups composed of patients and healthy controls under 45 years and subgroups composed of patients and healthy controls over 45 years. The subgroups including patients and healthy controls under 45 years consisted of 18 female [mean?±?SD age, 31.9?±?7.86?years] and 5 male [39.6?±?4.51?years] PXE patients as well as 18 female [32.0 ± 7.87 years] and 5 male [39.8 ± 4.60 years] blood donors as healthy controls. The subgroups including patients and healthy controls over 45 years consisted of 14 female [55.0?±?8.20?years] and 8 male [54.6?±?9.38?years] PXE patients as well as 14 female [54.5 ± 6.65 years] and 8 male [53.6 ± 6.70 years] blood donors as healthy controls.

### Cell culture

Normal human dermal fibroblasts (NHDF) from healthy controls were purchased from Coriell Institute for Medical Research (Camden, USA). Dermal fibroblasts from PXE patients were isolated from skin biopsies [32]. A summary of fibroblast characteristics used can be additionally found in Table 1.

For cultivation, Dulbecco’s modified essential medium (DMEM, Gibco, Invitrogen, Germany) containing 10% fetal calf serum (FCS; Biowest, Aidenbach, Germany), 2% L-glutamine (200mM) (PAN Biotech, Eidenach, Germany) and 1% antibiotic/ antimycotic solution (PAA Laboratories, Pasching, Austria) was used. Cells were subcultured as they reached confluency.

For experiments cells between passage 9 and 12 were used. Biological samples were performed in triplicates for all experiments. Cells were seeded with a final density of 177 cells/ mm^2^ using 60 mm culture dishes (BD Falcon) and cultivated for 24 h in 10% FCS. After this, cells were washed with phosphate-buffered saline (PBS; Gibco, Invitrogen, Germany) and medium was replaced with fresh 10% FCS. Medium was changed every 3-4 d and cells were cultured for 21 d.

**Table 1:** Characterization of human dermal fibroblasts from PXE patients and healthy controls.

Sample ID	Gender	Age^1^	Biopsy source	*ABCC6* genotype^2^		Genotype status
*PXE patients*						
P3M ^a^	male	57	Neck	c.3421C>T (p.R1141X)	c.3883-6G>A (SSM)	cht
P128M ^a^	male	51	Neck	c.3769_3770insC (p.L1259fsX1277)	c.3769_3770insC (p.L1259fsX1277)	hm
P255F ^a^	female	48	Arm	c.3421C>T (p.R1141X)	c.2787+1G>T (SSM)	cht
*Healthy controls*						
M57A ^b^ (AG13145)	male	57	Arm	-	-	wt
M52A ^b^ (AG11482)	male	52	Arm	-	-	wt
F48A ^b^ (AG14284)	female	48	Arm	-	-	wt

hm, homozygote; cht, compound heterozygote; ht, heterozygote; wt, wild type; SSM, splice site mutation. ^a ^Fibroblasts isolated from skin biopsies [32] . ^b ^Fibroblasts purchased from Coriell Institute for Medical Research (Camden, USA). ^1^Age in years. ^2^Nucleotide numbering refers to the cDNA sequence with the A of the ATG translation initiation start site as nucleotide +1 (GenBank accession number NM_001171.2).

### Nucleic acid isolation

RNA isolation was performed using the NucleoSpin RNA Kit (Macherey-Nagel, Düren, Germany), according to the manufacturer’s instructions. For DNA isolation, a 50 µl aliquot was collected after the first column based cleanup step. DNA isolation was performed using NucleoSpin Blood extraction Kit (Macherey-Nagel, Düren, Germany). DNA concentrations were used to normalize ELISA measurement data of cell culture supernatants to the number of cells during culture.

### Gene expression analysis

RNA was isolated as previously described. 1 µg RNA was transcribed to cDNA using SuperScript II Reverse Transcriptase (Thermo Fisher Scientific, San Diego, USA). For final measurements 2.5 µl cDNA (1:10), 0.25 µl forward and reverse primer (Biomers, Ulm, Germany), 2.0 µl water and 5.0 µl LightCycler 480 SYBR Green I Master reaction mixture (Roche, Penzberg, Germany) was mixed. After incubation for 5 min at 95°C, gene expression was determined by performing 45 cycles of denaturation (95°C, 10 s), annealing (specific annealing temperature, 15 s) and elongation (72°C, 20 s). Thereafter melting curve analysis was done. Quantitative real-time PCR (qPCR) was performed using LightCycler 480 (Roche, Penzberg, Germany) under conditions described in Table 2. As IGFBP3 and GDF11 showed aberrant or ambiguous serum concentrations, relative mRNA expression levels were measured and normalized to relative β-actin (*β-ACTIN*), glyceraldehyde-3-phosphate dehydrogenase (*GAPDH*) and β2-micro-globulin (*β2M*) mRNA expression. Results were calculated using delta-delta Ct method considering PCR efficiency. Technical triplicates were performed for each biological sample.

### Quantification of aging biomarker in human sera and cell culture supernatants

Concentrations of GDF11, IGF1, IGFBP3 and CCL11 were measured in sera from 45 PXE patients and sera from 45 age /sex-matched healthy controls. For IGF1, IGFBP3 and CCL11 Human Quantikine ELISA Kits (R&D Systems, Abingdon, UK) were used according to manufacturer’s instructions.

For GDF11 a DuoSet ELISA Development Kit (R&D Systems, Abingdon, UK) was used to develop a sandwich ELISA. The GDF11 ELISA was basically designed as recommended by the manufacturer. GDF11 standard was set up in FCS as calibrator diluent. For background measurement of standard, the zero standard was used. To consider any interfering effects of human sera on the antibody reaction, the lowest sample value measured was taken as background measurement [33].

For targets showing ambiguous results (GDF11) or aberrant serum concentrations in PXE patients compared to controls (IGFBP3) additional mRNA analysis in fibroblasts and protein determination in cell culture supernatants was performed when possible. Measurements of GDF11 in cell culture supernatants failed because concentrations were below detection limit (31.3 pg/ml).

### Statistical analysis

For qPCR analysis and protein concentrations of supernatants, data are shown as means ± standard error (SEM). For serum protein concentrations, data are shown as Box-Plot with median, 25^th^ and 75^th^ percentile and Tukey whiskers (± 1.5 times interquartile range). GraphPad Prism 5.0 was used as a statistical software. Data distribution was analyzed by Shapiro-Wilk test. For results of the measurement of serum concentrations a Student’s t-test (unpaired, two-tailed) was performed in case of normal distribution. If one of the compared groups was not Gaussian distributed the non- parametric two-tailed Mann-Whitney U test was done. For gene expression results and protein concentrations in supernatants the non- parametric two-tailed Mann-Whitney U test was performed. P-values of 0.05 or less were considered statistically significant.

**Table 2:** Primer sequences used for quantitative real-time PCR.

Gene	Protein	5´-3´sequence	Reference^1^	Annealing temperature (°C)	Efficiency
*ß-ACTIN* *beta-Actin*	ß-Actin	CGCGAGAAGATGACCC ATTGCCAATGGTGATGAC	NM_001101	59°C	2.0
*GAPDH* *glyceraldehyde-3-phosphate dehydrogenase*	GAPDH	AGGTCGGAGTCAACGGAT TCCTGGAAGATGGTGATG	NM_002046	59°C	1.8
*β2M* *beta-2-microglobulin*	ß2M	TGTGCTCGCGCTACTCTCTCTT CGGATGGATGAAACCCAGACA	NM_004048	59°C	2.0
*GDF11* *growth differentiation factor 11*	GDF11	AGGCCATTGGCAGAGCATCGAC GTCCCAGCCGAAAGCCTCAAAG	NM_005811.3	63°C	2.0
*IGFBP3* *insulin-like growth factor binding protein 3*	IGFBP3	GCGCCAGGAAATGCTAGTGA GGGAATGTGTACACCCCTGG	NM_001013398.1	63°C	1.8

1 Accession numbers from reference sequences taken from GenBank are shown.

## RESULTS

### Increased serum concentrations of CCL11 in aged individuals

**Figure 1.**
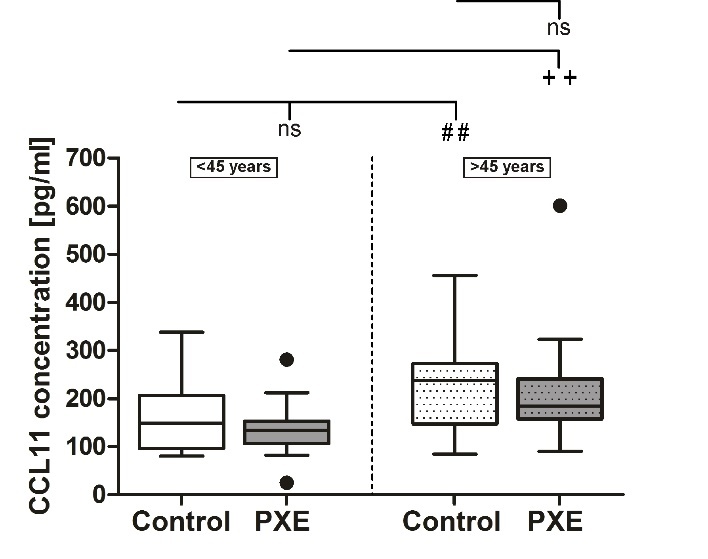
**CCL11 protein concentration in sera from PXE patients (grey) and healthy controls (white).** Data are shown as Box-Plot with median, 25^th^ and 75^th^ percentile and Tukey whiskers (± 1.5 times interquartile range). Control vs. PXE: ns p>0.05. Cohorts <45 years (n=23) vs. cohorts >45 years (n=22): ##/++ p≤0.01.

As seen in Fig. 1, means of serum concentration of CCL11 increased significantly with age in PXE patients (<45 years: 135.4 ± 10.6 pg/ml; >45 years: 207.8 ± 22.9 pg/ml; p≤0.01) as well as in healthy controls (<45 years: 158.9 ± 13.0 pg/ml; >45 years: 231.7 ± 21.8 pg/ml; p≤0.01). No significant differences in CCL11 protein concentration were seen between PXE patients and healthy controls.

### Aberrant serum concentration and decreased gene expression of GDF11 in PXE patients

**Figure 2.**
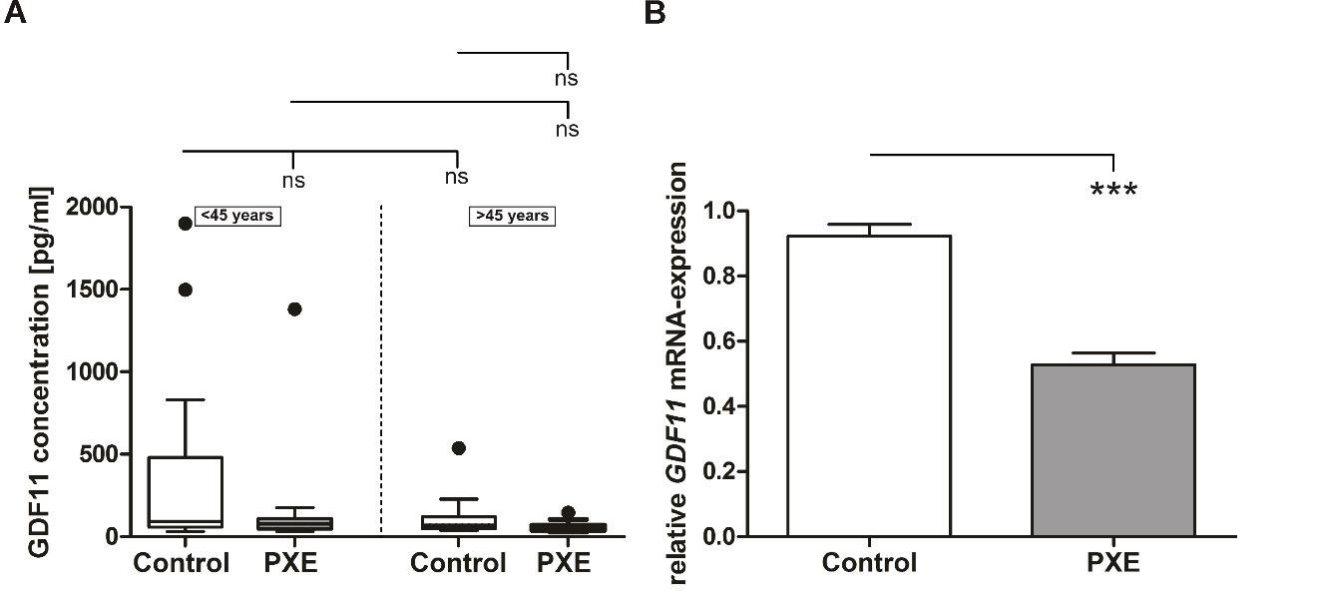
**Systemic concentration and local mRNA expression of GDF11. (A)** GDF11 protein concentration in sera from PXE patients (grey) and healthy controls (white). Data are shown as Box-Plot with median, 25^th^ and 75^th^ percentile and Tukey whiskers (± 1.5 times interquartile range). **(B)** Relative *GDF11* mRNA-expression of PXE fibroblasts (grey) and NHDF (white). Data are shown as mean ± SEM. Control vs. PXE: *** p ≤0.001. Cohorts <45 years (n=23) vs. cohorts >45 years (n=22): ns p>0.05.

Because of low GDF11 serum concentrations, 20% of measured values were outside standard curve and were not included into further data analysis. However, none of the findings reached statistical significance due to the high variances among individuals (Fig. 2A).

To further evaluate GDF11 levels in PXE patients, mRNA expression analysis was performed. As seen in Fig. 2B, *GDF11* mRNA expression in PXE fibroblasts was significantly decreased compared to age/sex-matched NHDF (PXE: 0.53 ± 0.04; control: 0.92 ± 0.04; p≤0.001). Determination of GDF11 protein concentrations in supernatants was not possible because concentrations were below the detection limit.

### Altered protein concentration and gene expression of IGF1 and IGFBP3

**Figure 3.**
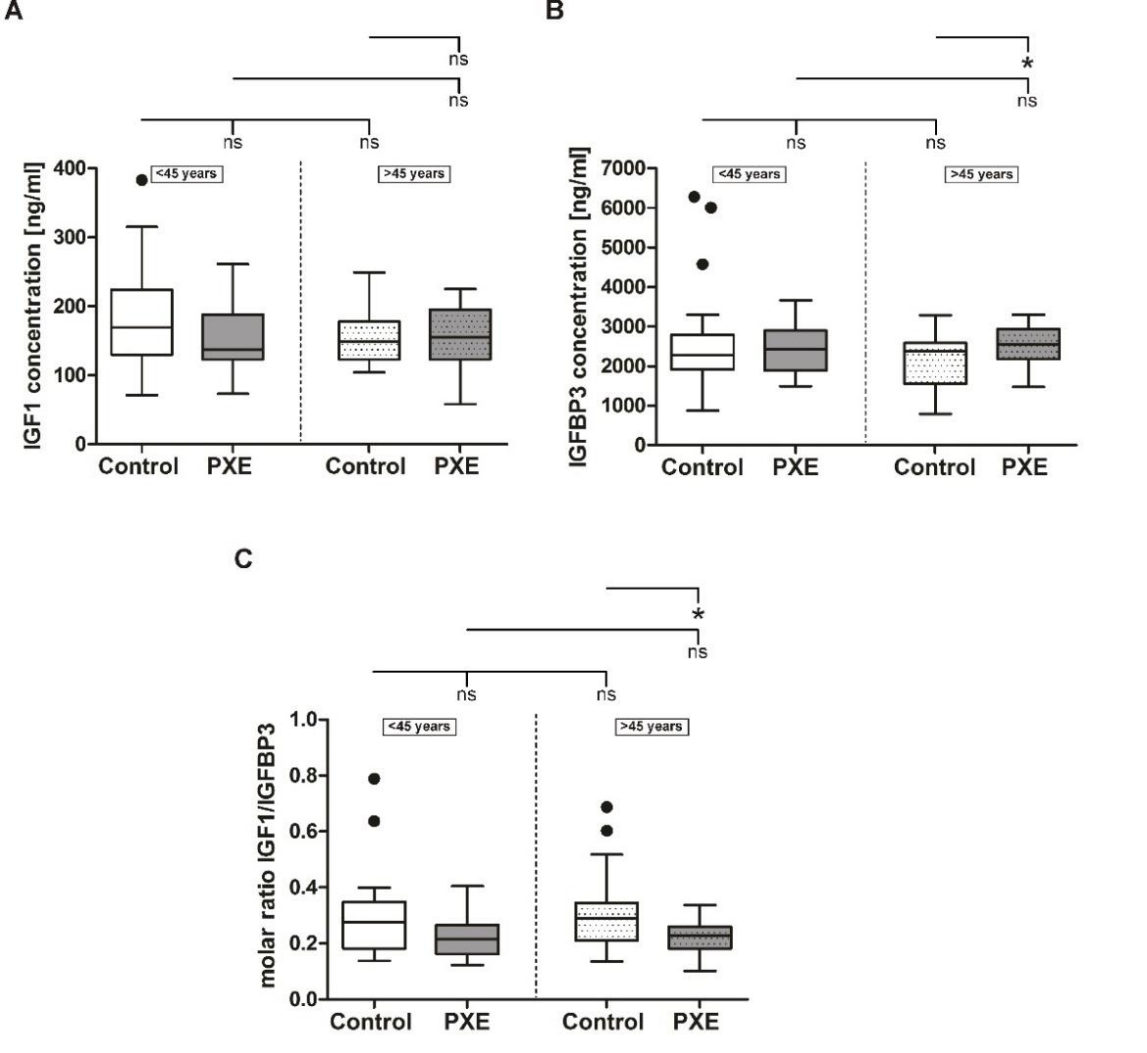
**Systemic IGF1 and IGFBP3 protein concentration in sera from PXE patients (grey) and healthy controls (white). (A)** IGF1 serum protein concentrations of PXE patients (grey) and healthy controls (white) **(B)** IGFBP3 serum protein concentrations of PXE patients (grey) and healthy controls (white) **(C)** molar IGF1/IGFBP3 ratio of serum protein concentrations of PXE patients (grey) and healthy controls (white). Data are shown as Box-Plot with median, 25^th^ and 75^th^ percentile and Tukey whiskers (± 1.5 times interquartile range). Control vs. PXE: * p≤0.05; ns p>0.05. Cohorts <45 years (n=23) vs. cohorts >45 years (n=22): ns p>0.05.

In healthy controls, serum protein concentration of IGF1 showed a minor decrease with age. This was not seen for serum concentrations of IGF1 in PXE patients. However, in PXE patients under 45 years slightly lower IGF1 levels compared to the appropriate healthy control group were observed. None of these results reached statistical significance (Fig. 3A).

For IGFBP3, no significant age dependent changes in serum concentrations were seen for PXE patients or for healthy controls. However, PXE patients over 45 years showed a significant increase in serum IGFBP3 concentrations compared to appropriate healthy controls (PXE: 2543.0 ± 104.9 ng/ml; control: 2117.0 ± 147.6 ng/ml; p≤0.05) (Fig. 3B).

Consequently, the molar IGF1/IGFBP3 ratio showed no differences with age but a significant decrease in PXE patients over 45 years compared to appropriate healthy controls (PXE: 0.22 ± 0.01; control: 0.31 ± 0.03; p≤0.05) (Fig. 3C).

**Figure 4.**
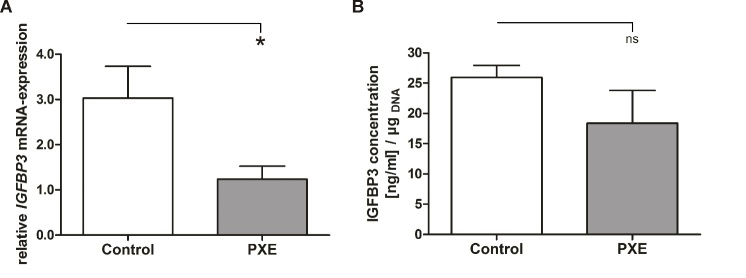
**Local IGFBP3 mRNA expression and protein concentration. (A)** Relative *IGFBP3* mRNA expression of PXE fibroblasts (grey) and NHDF (white). **(B)** IGFBP3 protein concentration in supernatant of PXE fibroblasts (grey) and NHDF (white). Data are shown as mean ± SEM. Control vs. PXE: * p≤0.05; ns p>0.05.

**Figure 5.**
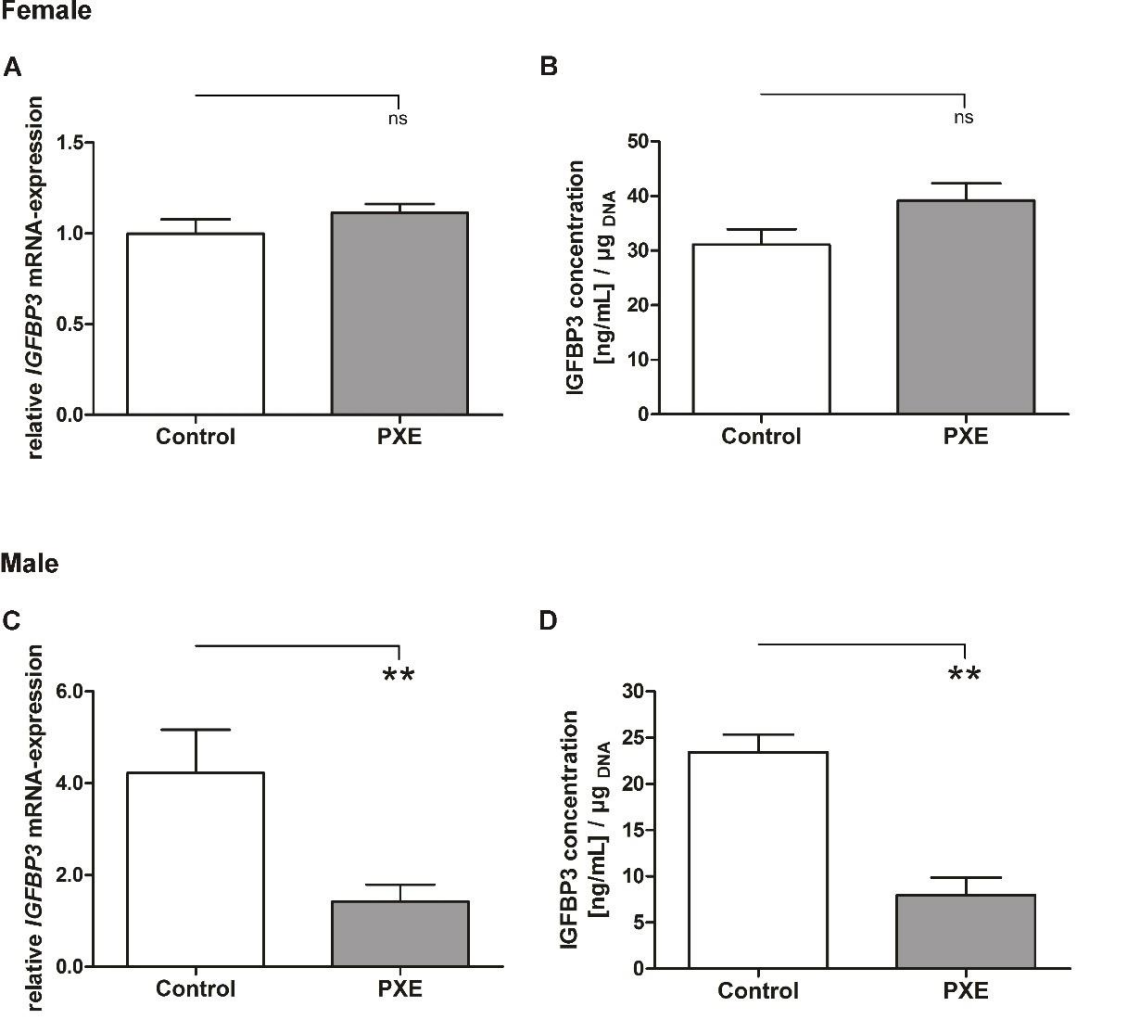
**Male and female specific IGFBP3 mRNA expression and protein concentration. (A)** Relative *IGFBP3* mRNA expression of female PXE fibroblasts (grey) and NHDF (white). **(B)** IGFBP3 protein concentration in supernatant of female PXE fibroblasts (grey) and NHDF (white). **(C)** Relative *IGFBP3* mRNA expression of male PXE fibroblasts (grey) and NHDF (white). **(D)** IGFBP3 protein concentration in supernatant of male PXE fibroblasts (grey) and NHDF (white). Data are shown as mean ± SEM. Control vs. PXE: ** p≤0.01; ns p>0.05.

As IGFBP3 showed aberrant protein concentrations in PXE sera, we performed qPCR analysis for determination of gene expression in PXE fibroblasts compared to NHDF. As seen in Fig. 4A, PXE fibroblasts showed a significant decrease in *IGFBP3* mRNA expression compared to NHDF (PXE: 1.24 ± 0.29; control: 3.03 ± 0.70; p≤0.05). We further showed a decrease of IGFBP3 protein concentration in supernatants of PXE fibroblasts compared to NHDF which did not reach statistical significance (Fig 4B). This was mainly due to potential gender specific variations. As seen in Fig. 5A-B, *IGFBP3* mRNA expression as well as protein concentration of supernatant showed no differences in PXE fibroblasts compared to NHDF for females. In contrast to this, *IGFBP3* mRNA expression (PXE: 1.42 ± 0.37; control: 4.23 ± 0.94; p≤0.01) and protein concentration of supernatants (PXE: 8.0 ± 1.9 [ng/mL]/µg_DNA_; control: 23.4 ± 1.9 [ng/mL]/µg_DNA_; p≤0.01) were significantly decreased in PXE fibroblasts compared to NHDF for males (Fig. 5C-D).

### DISCUSSION

PXE is a rare inherited genetic disorder mainly caused by mutations in the *ABCC6* gene [5, 6]. By now, PXE pathogenesis has just barely been discussed in the view of the fact that the clinical and molecular characteristics of PXE patients equal those seen in premature aging and premature aging syndromes. Thus, this study was performed to evaluate specific aging biomarkers in sera and fibroblasts from PXE patients to get insights into potential aging processes associated with an ABCC6 deficiency in PXE patients.

The first biomarker analyzed is CCL11. CCL11 is a chemokine, acting as a chemoattractant for eosinophils and plays a role in arteriosclerosis, inflammation and neurogenesis [29, 34, 35]. We found significantly increased serum concentrations of CCL11 with age in both, PXE patients and healthy controls. This is in accordance with previous studies showing an increase of serum CCL11 concentration with age [34, 36]. Furthermore, our study showed no significant differences in CCL11 serum concentrations between PXE patients and healthy controls. Elevated CCL11 serum concentrations are associated with different stages of age-related macular degeneration (AMD), except for neovascular AMD. Patients with neovascular AMD showed, like PXE patients in our study, normal CCL11 serum concentrations compared to healthy controls [37]. This matches the fact that the vision impairments of PXE patients show similarities like choroidal neovascularization (CNV) with neovascular AMD [7, 38-40].

Another intensively discussed aging biomarker is GDF11, a member of the transforming growth factor-β superfamily and closely related to myostatin (GDF8). GDF11 plays a role in mammalian development and was claimed to have anti-aging effects [22, 24, 41]. We have seen a minor decrease in GDF11 serum concentrations with age and between PXE and healthy controls, though these changes did not reach statistical significance. Previous studies investigating age dependent GDF11 blood level showed controversial results possibly due to specifity and selectivity of detection methods and to potential low overall GDF11 serum concentration [24, 33, 42-44]. Although, there was not always strictly distinguished between GDF8 and GDF11, some studies reported an overall decrease of GDF11 or GDF11/8 concentrations in the bloodstream with increasing age [24, 44]. Loffredo et al. showed a reduction in GDF11 serum concentrations and proved that recombinant GDF11 can reverse age-related cardiac hypertrophy in mice [24]. In 2015, a study by Olsen et al. confirmed this by linking decreasing GDF11/8 levels to higher risks of cardiovascular events and death [45]. As we could not detect any significant changes in GDF11 serum concentrations for PXE patients, it could be suggested that circulating GDF11 plays only a minor role in PXE or age-related pathogenesis going along with it. These ambiguous results rather raised the question whether local and not systemic GDF11 concentrations are relevant in PXE. We, therefore, measured *GDF11* mRNA expression in human dermal fibroblasts from PXE patients as well as in NHDF and found a significant reduction in mRNA expression in PXE fibroblasts compared to NHDF. Unfortunately, determination of GDF11 levels in cell culture supernatants was not possible due to low protein concentrations. However, previous studies showed a decreased *GDF11* mRNA and protein expression in spleen could be associated with age in mice [24]. It was proposed that *GDF11* expression in spleen is rather high compared to other tissues and may primarily contribute to circulating GDF11 concentrations [24]. Thus, the decreased *GDF11* mRNA expression in PXE fibroblasts do not necessarily correlate with detected circulating GDF11 levels. This probably strengthen the assumption that autocrine or paracrine effects of aberrant locally expressed GDF11 levels could be more important for PXE pathogenesis in affected tissues than circulating GDF11.

A third protein which is closely associated with aging is IGF1. IGF1 plays a wide role in proliferation and tissue growth and is known as an anti-aging factor ([30], reviewed in [46]). IGF1 serum levels peak in young adolescents and subsequently declines with age [47]. Studies showed that progeroid *Zmpste24^-/-^* mice have a disturbed IGF1/growth hormone (GH) ratio with low IGF1 plasma concentrations. Application of recombinant IGF1, normalized IGF1/GH ratio and extended the lifespan of *Zmpste24^-/-^* mice [30].

Measuring total serum IGF1 concentrations in PXE patients and controls, we found no statistical significant changes with age as the variances of individuals in each group may mask the expected age associated decline of total IGF1 levels. Apart from this no significant differences were seen between PXE and control cohorts. As the main source for circulating IGF1 is the liver [48] direct effects of ABCC6 deficiency on expression and secretion of it are unlikely.

The majority of circulating IGF1 is bound to IGFBP3 which strictly controls IGF1 availability (reviewed in [49]). Additionally, studies showed an increase in IGFBP3 expression in old NHDF as well as in senescent human umbilical vein endothelial cells compared to young non senescent cells [28, 50]. We found that IGFBP3 serum levels in PXE and control sera showed no significant difference with age, but a significant increase in PXE patients over 45 years compared to the appropriate control cohort. By calculating the molar IGF1/IGFBP3 ratio it could be suggested that less circulating free IGF1 is present in PXE patients over 45 years compared to healthy controls. This possibly results in a reduced IGF1 signaling in peripheral tissues which supports the hypothesis of an accelerated aging process in PXE patients.

In addition, studies also showed that locally expressed IGFBP3 may have paracrine or autocrine effects on tissue specific IGF1 signaling as local IGFBP3 expression can differ from circulating serum concentrations [51]. We measured *IGFBP3* mRNA expression and protein concentration in supernatants of primary human dermal fibroblasts of PXE patients and healthy controls to evaluate tissue specific IGFBP3 expression. Both, mRNA expression and protein analysis showed an overall decrease in PXE fibroblasts compared to NHDF. Interestingly, mRNA expression and protein concentrations in supernatants showed opposed results in males and females with decreased expression in males and no changes for females. One has to mention that the fibroblasts from male PXE donors are derived from different skin areas as the control fibroblasts and the fibroblasts from the female PXE donor. Thus, instead of gender associated reasons, the observed opposed results could, thus, be also derived from different expression patterns due to the biopsy source. Nevertheless, results from Oliver et al. support the gender specific expression of IGFBP3 as they showed decreased IGFBP3 protein expression in male and even no change of protein expression in female IGF1 overexpressing skeletal muscle of old mice compared to younger ones [51]. They also showed that this sexual dimorphism was not due to a different response to locally overexpressed IGF1 but to alternative age and gender dependent factors [51]. Contrary to their results, we did not only see differences in protein concentration but also distinct mRNA expression between genders which could be related to tissue specific regulation. Thus, we see a decreased *IGFBP3* mRNA expression in PXE fibroblasts of males and no significant changes in female PXE fibroblasts compared to NHDF. A study of Lovqist et al. showed increased *IGFBP3* mRNA expression in retinal vasculature under conditions of hypoxia [52]. They assumed a protective function of IGFBP3 contributed to the prevention of oxygen-induced vessel loss and abnormal neovascularization [52]. Low tissue specific IGFBP3 concentrations could, possibly, promote CNV when also found in retinal tissue. CNV is a prominent clinical manifestation seen in PXE patients [7, 8, 39]. Surprisingly, the observed sexual dimorphism showed only low tissue specific IGFBP3 expression in males although it is often reported that females are predominantly affected by PXE [7]. At first sight, the results for tissue specific expression did not match the results obtained by the measurement of serum IGFBP3 levels showing increased IGFBP3 concentrations for PXE patients compared to healthy controls. As far more females than males were tested for IGFBP3 serum levels this may explain why an increase rather than a decrease was seen for IGFBP3 serum concentrations. Additionally, the other tested biomarkers showed no gender specific expression patterns which demonstrated that this potential sexual dimorphism probably only applies for selected targets and is not a general phenomenon.

Nevertheless, our results show that ABCC6 deficiency may lead to aberrant IGFBP3 mRNA and protein expression in PXE fibroblasts associated with potential gender specific aging processes, again, strengthen the assumption of an accelerated aging process in PXE patients. Furthermore, it underlines that an ABCC6 deficiency has a direct influence on affected peripheral tissues.

This is the first study linking ABCC6 deficiency to aberrant systemic and especially local concentrations of aging biomarkers in peripheral tissues. These findings could be associated with a potential premature aging process in PXE patients and particularly in affected peripheral tissues. As a result of this, it should be considered that clinical manifestation seen in PXE patients, like visual impairments or soft tissue calcification which are, thusfar, claimed to be direct results of aberrant concentrations of the unknown ABCC6 substrate, could also be indirect consequences caused by premature aging processes induced by e.g. an aberrant lipid metabolism due to ABCC6 deficiency [53].
